# Visuo-motor and interoceptive influences on peripersonal space representation following spinal cord injury

**DOI:** 10.1038/s41598-020-62080-1

**Published:** 2020-03-20

**Authors:** Michele Scandola, Salvatore Maria Aglioti, Giovanna Lazzeri, Renato Avesani, Silvio Ionta, Valentina Moro

**Affiliations:** 1 NPSY-Lab.VR, Department of Human Sciences, University of Verona, Verona, Italy; 2 IRCCS, Fondazione Santa Lucia, Rome, Italy; 3 Centro Giusti, Florence, Italy; 4 Department of Rehabilitation, IRCSS Sacro Cuore - Don Calabria Hospital, Verona, Italy; 5 Department of Psychology, University of Rome “Sapienza”, Rome, Italy; 6 Sensory-Motor Lab (SeMoLa), Department of Ophthalmology-University of Lausanne, Jules Gonin Eye; Hospital-Fondation Asile des Aveugles, Lausanne, Switzerland; 7 Istituto Italiano di Tecnologia, Rome, Italy

**Keywords:** Perception, Perception, Human behaviour, Human behaviour

## Abstract

Peripersonal space (PPS) representation is modulated by information coming from the body. In paraplegic individuals, whose lower limb sensory-motor functions are impaired or completely lost, the representation of PPS around the feet is reduced. However, passive motion can have short-term restorative effects. What remains unclear is the mechanisms underlying this recovery, in particular with regard to the contribution of visual and motor feedback and of interoception. Using virtual reality technology, we dissociated the motor and visual feedback during passive motion in paraplegics with complete and incomplete lesions and in healthy controls. The results show that in the case of paraplegics, the presence of motor feedback was necessary for the recovery of PPS representation, both when the motor feedback was congruent and when it was incongruent with the visual feedback. In contrast, visuo-motor incongruence led to an inhibition of PPS representation in the control group. There were no differences in sympathetic responses between the three groups. Nevertheless, in individuals with incomplete lesions, greater interoceptive sensitivity was associated with a better representation of PPS around the feet in the visuo-motor incongruent conditions. These results shed new light on the modulation of PPS representation, and demonstrate the importance of residual motor feedback and its integration with other bodily information in maintaining space representation.

## Introduction

Acquired spinal cord injuries (SCIs) typically cause varying degrees of impairment in sensory and motor functions in the body parts below the lesion, potentially also involving complete paralysis and anaesthesia.

To date, the impact of sensorimotor disorders on cognitive processing has been for the most part neglected or only tested in patients with brain lesions^1–3^. However, if research is limited to studies on these patients, it means that it is not possible to distinguish between the effects relating to brain damage and those relating to motor deefferentation and somatosensory deafferentation. Thus, in order to shed light on the role of sensorimotor information in cognition, it is crucial to test cognitive modifications in patients with sensorimotor impairments but without brain damage, a condition that is typical of SCI patients. An increasing number of reports have focused on the effects of SCI on body- and action-related cognitive representations, including changes in action representation^4–6^, motor learning^7,8^, motor imagery^9,10^ and body representation^11–18^. Interestingly, these studies indicate that SCI induces changes that, in terms of cognitive representation, may lead to both an increase in the functions of body parts above the neurological lesion level (i.e. the most caudal segment of the spinal cord with normal sensory and motor functions on both sides of the body^19^) and a decrease in functions below the neurological lesion level^6^.

For example, in cases of SCI, Peripersonal Space (PPS) (i.e. “a particularly relevant sector of space, as it is where all physical interactions between the individual and the environment take place.”^20^ p. 139) is reduced around the body parts below the lesion level (e.g. around the feet), but preserved around the body parts above the lesion level (e.g. the hands)^21^. Even if only few studies have focused on PPS around the feet, these indicate its presence in healthy subjects^21–24^.

The so-called Crossmodal Attention Paradigm^25^ is a sensitive task which has been widely used to estimate PPS objectively. Schicke and colleagues^22^ proposed a modified version to estimate the PPS around the feet.

The participants in the Crossmodal Attention Paradigm held two tactile target stimulators, one in each hand. These could both vibrate independently either against the index finger (upper elevation) or against the thumb (lower elevation). Distractor lights were fitted onto a wooden frame for the feet. The participant’s feet were placed inside two compartments within the wooden frame, and the 4 distracter lights were placed near the lower or the upper elevation (sole or the upper part of the feet) of both feet.

The tactile and visual stimuli were presented almost simultaneously. The task involved judging the location of the tactile stimulus while ignoring the visual stimulus (i.e. the distractor lights). The stimuli were either congruent (i.e. with the tactile stimulus on the index finger and the distractor light on the upper part of the foot or incongruent (i.e. with the tactile stimulus on the thumb and the distractor light on the upper part of the foot). The difference in reaction times to the tactile stimuli in the congruent as compared to the incongruent condition is known as the Crossmodal Congruency Effect (CCE). The CCE is greater when the visual stimuli are within the participant’s PPS and smaller when they are outside the PPS^26,27^. A similar pattern of results emerges when the tactile and visual stimuli are on the same body side in comparison with stimuli that are on the opposite side of the body^28^. In fact, the two stimuli presented on the same side are processed as being inside the PPS around that body part, while when these are on the opposite side (e.g. a tactile stimulus on the left hand and a visual stimulus on the right foot) the visual stimulus is processed as being outside the PPS of that body part^22,29,30^.

The CCE paradigm^22^ was previously used by us in a study on SCI participants with complete paraplegia^21^.

The results of that study indicated that there was a shrinkage in the representation of PPS around the feet of the participants affected by SCI and a normally preserved representation of hand-related PPS^21^ suggesting that the lack of sensory and motor functions affecting certain specific body parts impacts the representation of the space surrounding those parts. Significantly, the same study also showed that 15 minutes of passive leg mobilisation were enough to restore the PPS around the feet. However, to date, the mechanisms underlying the recovery of PPS remain unexplained.

A variety of factors, ranging from feedback relating to the deafferented/deefferented body parts to interoception (i.e. a person’s sense of the physiological condition of their body^31^), may be at play. For example, the recovery may be influenced by visual feedback relating to the body. Indeed, in the previously mentioned study concerning PPS in SCI^21^, passive mobilisation was administered while the participants were looking at their legs. This visual input might thus induce a sort of “illusion” of movement that in some way impacts on the individual’s PPS representation. Another hypothesis is that in the absence of below-lesion afference, somatic sensations coming from the body parts above the lesion level become particularly developed in cases of SCI. This may help individuals to rebuild their body and around-body representations during mobilisation.

Interoception might also play a role in PPS recovery. In complete paraplegics, interoception may be the only spared sensory function potentially able to connect deafferented body parts with a general body representation. Interestingly, people with SCI often report that they are particularly sensitive in terms of their perception of specific body states (e.g. the bladder needing to be emptied, excessive pressure on the body parts resting on the wheelchair). This is based on sensory signals coming from body parts above the lesions (e.g. shivers on the neck, headache). When associated with passive movement, the contribution of interoception to body awareness^31–33^ may potentially mediate the recovery of PPS around the feet.

The aim of this study is to comprehend the mechanisms that underlie the recovery of PPS following passive mobilisation. All of the abovementioned components (i.e. visual, residual upper lesion somatosensory and interoceptive information) are therefore considered. Our approach may contribute towards a better understanding of the role of the body in cognition and the interaction between bodily information and space representation.

By means of Immersive Virtual Reality (IVR), we aimed to dissociate the impact of Motion and Visual information during passive mobilisation. In order to do this, four conditions were considered, recorded and analysed. These were related to whether the motor and visual information that the participants received was congruent or non-congruent: 1 - Vision: Mobilisation, Motion: Mobilisation; 2 - Vision: Mobilisation, Motion: No Mobilisation; 3 - Vision: No Mobilisation, Motion: Mobilisation and 4 – Vision: No Mobilisation, Motion: No Mobilisation. For each condition, data from the PPS assessment were compared to the PPS results in the baseline and follow-up assessments. Furthermore, the influence of autonomic, interoceptive signals and interoceptive awareness were recorded and considered in the main analyses. The experiment was divided into two sessions. In Session 1, we investigated the effects of showing a video in which the participant could see a pair of legs being passively mobilised (Vision: Mobilisation), either accompanied by actual passive movement (Motion: Mobilisation) of their legs or without any actual movement (Motion: No Mobilisation). In Session 2, we studied the effects of the participant being shown a pair of immobile legs (Vision: No Mobilisation) either with the passive mobilisation of the participant’s legs (Motion: Mobilisation) or without (Motion: No Mobilisation) Mobilisation. Behavioural and physiological measures were recorded in both sessions.

## Methods

### Participants

The participants were divided into three groups: i) people who had suffered SCI with Complete lesion and Paraplegia (CP); ii) people who had suffered SCI but with Incomplete lesion and Paraplegia (IP) and iii) a group of gender and age matched healthy controls (Control group, C).

**Figure 1:**
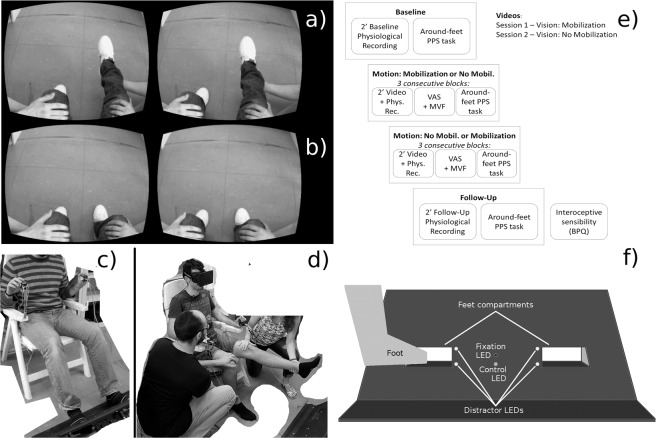
The experimental set-up and the timeline for Experiments 1 and 2. (**a**) Picture from the Vision: Mobilisation video. (**b**) Picture from the Vision: No Mobilisation Video. (**c**) Position of the participants during the CCE evaluation. (**d**) Position of the participant and of the experimenters during the observation of a Video. In this particular case, the experimenters were applying manual passive motion (Motion: Mobilisation). (**e**) General timeline of the experiments. Vision: Mobilisation stands for the visualization of a video showing passive motion (represented in this Figure in a), Vision: No Mobilisation stands for the visualization of a video showing still legs in a resting position (represented in this Figure in b). Motion: Mobilisation stands for the application of manual passive motion (represented in this Figure in d). Motion: No Mobilisation stands for no application of passive motion. (**f**) Schematic representation of the frontal part of the wooden frame used for the visual stimuli around the feet. The representation of the foot in the image is not in an anatomical position (during the experiment the participants were in front of the wooden frame) but this gives a better idea of where the participant’s feet were placed. VAS = Visual Analogue Scale; BPQ = Body Perception Questionnaire; MVF = Movement Verbal Feedback.

There were 19 CP participants in total (4 females; mean age = 45.05, SD = 12.04), with 9 of them taking part in both sessions. The IP group comprised a total of 23 participants (9 females; mean age = 46.39, SD = 13.50), with 5 of them taking part in both sessions. The clinical and demographic data of the SCI participants are shown in Table 1. 28 controls (C) took part, 14 for each session (4 females in each experiment; mean age = 41.41, SD = 11.50). We tested a total of 70 participants. While 16 participants participated in both of the two experimental sessions, the remaining 54 participants only took part in one session. Those participants who took part in both sessions had a wash-out period of between 81 and 257 days (mean (SD) 169 (50)), thus excluding any learning effects. In each session, each group was composed of 14 participants. All of the participants were informed about the experimental procedure and had signed the relevant consent form. Signed consent was given by the participants and experimenters for the use of the photographs appearing in Fig. 1. All of the participants had normal or corrected-to-normal vision. The study was approved by the Ethics committee of the Province of Verona (Prot. N. 40378) and was conducted in accordance with the ethical standards of the 2013 Declaration of Helsinki.

**Table 1:** Clinical and demographic data of the SCI participants.

	Participant	Age	Gender	Session	AIS	NLI
Complete Paraplegics	01	25	M	Vision: No Mobilisation	A	T12
	02	42	M	Vision: No Mobilisation	A	T4
	03	46	F	Vision: No Mobilisation	A	T3
	04	54	M	Vision: No Mobilisation	A	T7
	05	63	M	Vision: No Mobilisation	A	T4
	06	31	M	Vision: Mobilisation	A	T3
	07	44	M	Vision: Mobilisation	A	T10
	08	51	M	Vision: Mobilisation	A	T4
	09	55	F	Vision: Mobilisation	A	T10
	10	62	F	Vision: Mobilisation	A	T7
	11	28	F	Both sessions	A	T3
	12	29	M	Both sessions	A	T6
	13	35	M	Both sessions	A	T6
	14	36	M	Both sessions	A	T4
	15	40	M	Both sessions	A	T4
	16	49	M	Both sessions	A	T11
	17	49	M	Both sessions	A	T7
	18	55	M	Both sessions	A	L3
	19	62	M	Both sessions	A	T4
Incomplete Paraplegics	20	25	M	Vision: No Mobilisation	B	T6
	21	30	M	Vision: No Mobilisation	B	T6
	22	35	M	Vision: No Mobilisation	B	T4
	23	45	M	Vision: No Mobilisation	D	L3
	24	52	M	Vision: No Mobilisation	D	L1
	25	52	F	Vision: No Mobilisation	D	L1
	26	53	M	Vision: No Mobilisation	D	T9
	27	21	F	Vision: Mobilisation	C	T6
	28	31	F	Vision: Mobilisation	B	T6
	29	37	M	Vision: Mobilisation	D	T6
	30	37	M	Vision: Mobilisation	D	T12
	31	40	M	Vision: Mobilisation	D	T12
	32	52	M	Vision: Mobilisation	D	L1
	33	57	F	Vision: Mobilisation	D	T7
	34	63	F	Vision: Mobilisation	D	L1
	35	64	F	Vision: Mobilisation	C	T6
	36	28	F	Both sessions	B	T6
	37	48	M	Both sessions	D	T12
	38	56	M	Both sessions	B	L2
	39	57	F	Both sessions	D	T7
	40	61	M	Both sessions	D	T5
	41	61	M	Both sessions	C	T11
	42	62	F	Both sessions	D	L1

Note: AIS = ASIA Impairment Scale; Note: AIS = ASIA Impairment Scale; A = complete lesion (no somatosensory or motor functions below the lesion level); B = spared sensory functions below the lesion level; C = spared sensory and motor functions below the lesion level, but no motricity against gravity; D = spared sensory and motor functions below the lesion level, with possibility of motion against gravity. NLI = Neurological Level of Injury^19^.

A power analysis was executed with a moderate effect size (Cohen’s f = 0.25) to achieve a power of 80%. The suggested sample size was 13.64.

### Materials

In order to test PPS, a pre-tested custom made apparatus was used^21^ (Fig. 1c). The apparatus consisted of a wooden frame (80 × 38 cm, tilted at 30° from the vertical plane) on which the four LEDs and the two compartments for the feet (each 15 × 8 cm, distance 22 cm) were positioned. The four white LEDs were placed in the proximity of the four inner corners of the foot compartments and these functioned as distractors. Two further LEDs (one red and one green) were placed in the centre of the wooden frame with the red light functioning as a fixation point and the green one as a control light. Tactile stimulation was administered by means of two stimulators which the participants held in their hands, with one stimulator in each hand (Dayton Audio DAEX25 Sound Exciter Pairs). These were small cylinders which could be made to vibrate independently at the contact points on either side, that is, the point of contact relating to the participants’ index finger and thumb.

A head mounted display (Oculus Rift DK1) was used to show the IVR videos. This was removed in the other phases. The videos were three-dimensional, filmed from an egocentric, first person perspective and lasted 2 minutes. One video (Vision: Mobilisation video, see Fig. 1a) showed a pair of legs (including the feet) which were being moved passively by two people with only their hands visible. One person moved the right leg and foot and the other person moved the left leg and foot for a duration of 2 minutes. Another video (Vision: No Mobilisation video, see Fig. 1b), which also lasted 2 minutes, showed the initial frame of the previous video with the legs immobile in a resting position and two people touching the legs but without moving them. In this way the social component of the stimuli due to the presence of other people remained constant in the two videos and the only difference was the presence or absence of passive movement. In order to record physiological signals, a MP150 system (BIOPAC Systems, Inc.) was used. This included: i) a GSR100C module to record Skin Conductance Levels (SCL) (the electrodes were positioned on the left shoulder); ii) a PPG100C module to record pulse pressure waves by means of photoplethysmography of the little finger and iii) a RSP100C module to record breathing frequency by means of a thoracic belt which detected expansion and contraction.

The participants in the Complete SCI group were unable to feel either their legs or feet both in the passive motion condition and in the immobile condition. Nevertheless, their sensitivity to their internal state might have resulted in alternative sensations related to the perception of motion. In order to check for this variable and for the participants’ subjective reactions to the experimental manipulation, the Body Awareness subscale of the Body Perception Questionnaire (BPQ^34^) was administered after the task. The BPQ is a self-reporting measure of body awareness and autonomic reactivity. The items refer to the autonomic nervous system and investigates the individual’s awareness of information coming from their body concerning the status of their organs and tissues (e.g. “I feel nauseous”; “I have joint pain”; “I have difficulty coordinating breathing with talking”; “I feel an urge to swallow”). This is designed to quantify a person’s individual interoceptive sensibility^35^. Items n. 12, 14 and 36 were removed since they investigate sensations which the CP participants were maybe unable to feel (i.e. the urge to urinate or defecate and the feeling of fullness in the bladder).

### Procedure

The timeline of the procedure is shown in Fig. 1e.

In Session 1 (Vision: Mobilisation), the participants sat in a comfortable position on a chair equipped with armrests and a reclining backrest (see Fig. 1c). The wooden frame was placed on the floor in front of them, and they were helped to place their feet in the two foot compartments positioned on the wooden frame. After a brief instruction session in which the participants were informed about the overall structure of the experiment and what they would see during the IVR videos, the Baseline block was initiated. The participants were requested to remain still but relaxed for 2 minutes while the SCL, pulse pressure waves and respiration signals were being recorded. A task which aimed to assess PPS around the participant’s feet was then executed (for details see^21^). In this task, the participants held a tactile stimulator in each hand, with the index finger and thumb of each hand positioned on the top and the bottom of the device respectively. Various different combinations of tactile stimulation and visual distractor were administered in each trial. The tactile stimuli (three 50 ms vibrations separated by 50 ms gaps, total duration 250 ms) were administered either to the upper or lower parts of the stimulation devices (i.e. index finger or thumb respectively) and on either the left or right stimulation device (i.e. the left or right hand). The visual distractor stimuli (3 × 50 ms separated by 50 ms gaps) were displayed close to one of the two foot compartments on the wooden frame, either on the upper or lower part of the compartment (upper: close to the dorsal part of the foot, lower: close to the plantar part of the foot, see Fig. 1f). In this way, there were four typologies of combination: i) Congruent (top-top or bottom-bottom) and Unilateral (left-left or right-right); ii) Congruent Bilateral (left-right or right-left); iii) Incongruent (upper - lower or lower - upper) and Unilateral and iv) Incongruent Bilateral. PPS representation influences the difference between the Incongruent Unilateral and Congruent Unilateral trials due to variations in the visual and tactile stimuli in the same portion of space, while in the Bilateral trials, the congruent/incongruent difference is considered to have a visual attentional effect^36^. The indexes obtained by means of these distinctions are known as Crossmodal Congruency Effects (CCE^28,29^). The CCE indexes are increased when the visual distractors placed near the feet and the tactile stimulation to the hands are administered on the same body side.

The participants were requested to look at the fixation LEDs on the wooden frame on the floor and, as quickly as possible, verbally report where they felt the tactile stimulus on their hand (i.e. “top” or “bottom”), irrespective of which hand the stimulus was administered to. They were told to ignore the visual distractors. The verbal response options had been selected with a view to avoiding the presence of fricative consonants in order to facilitate the participants’ enunciation thereby avoiding prolonged response times which would render the RT computation difficult. There are fricative consonants in the Italian words for top (“sopra”) and bottom (“sotto”) and thus the response options chosen were the sounds: i) “TAH” when the tactile sensation was felt on the index finger (top position) and ii) “TOH” when it was felt on the thumb (bottom position), irrespective of whether the stimulus was on the left or the right hand.

In order to ensure that the participants focused on the centrally positioned LEDs, they were instructed to say the word “luci” (the Italian word for “lights”) whenever they saw the green LED flashing (control trials), and to guarantee that they paid attention to the tactile stimuli, false stimulations were administered. These false stimulations consisted of a distractor light on the wooden frame which flashed without tactile stimulation. In these cases, the response was expected to be “niente” (the Italian word for “nothing”). The PPS task consisted of 56 trials in the Baseline and at the Follow-Up blocks (respectively, the first and the eighth block which was the last). No video was shown or any passive motion administered in these two conditions.

In the other blocks (i.e. from the second to the seventh block), during Session1 (Vision: Mobilisation), a head mounted display device showed a 2 minute video in which the participants watched a pair of legs being passively moved by two people. While they observed the video, the participants’ own legs (which they could not see) could either be synchronously moved (Motion: Mobilisation, 3 consecutive blocks, Fig. 1d), or kept immobile in a resting position (Motion: No Mobilisation, 3 consecutive blocks). During the video, the participants’ physiological signals were recorded. After each 2 minute video, the head mounted display was removed, and the participants were requested to answer a yes/no question concerning whether they had had the sensation that their legs were moving or that they had been still (Movement Verbal Feedback). This question was important in order for us to understand whether, notwithstanding the impaired body-brain connection, the CP and IP participants could correctly pick up some kind of residual information regarding the movement of their legs. After this, a PPS test was executed around their feet (36 trials).

The same procedure was used for Session 2 (Vision: No Mobilisation), with the difference that the video shown in the second to the seventh blocks depicted immobile legs (Vision: No Mobilisation video, see Fig. 1b). The order of the Motion: Mobilisation and Motor: No Mobilisation conditions in the two sessions and the order in which the participants took part in the two sessions were counterbalanced across participants. Finally, a Follow-Up block was executed, with the same procedure as the Baseline block (Fig. 1e).

### Data processing

Reaction times from the PPS task around the feet were extracted from audio files (WAV format at 8000 Hz) by means of a custom-made R^37^ script and the *tuneR* package^38^. A preliminary check of individual participants was made in order to ensure that all of them were able to achieve an accuracy level of >44% (i.e. greater than the chance level, excluding the control trials). This was done to ensure that all of the participants had understood the task correctly. Moreover, all the reaction times of less than 100 ms were removed since it takes at least 100 ms for human beings to detect a visual target^39^, or to detect a tactile stimulus to the hand while focusing on a foot^40^. Inverse Efficacy (IE) indexes were then computed by dividing reaction times by the proportion of correct trials for each block and each participant (proportions were used in order to take into account the varying number of trials between the baseline and follow-up and the other blocks). All the values outside the range [mean ± 2.5 SD] were considered outliers and were thus removed (parentheses indicate that the limit of the range is not included, while square brackets indicate that the limit of the range is included. For example, [0 ÷ 1) indicates a range between ≥0 and <1). The outliers constituted 3.12% of the original data.

The IEs were then used to estimate the CCE. In order to take into account any variances in the entire data set, we used a specific Bayesian model (see the Data Analysis section and the representation of the Data Analysis of PPS around the feet).

As the Movement Verbal Feedback was a binomial yes/no response, this was transformed to indicate accuracy (i.e. 1 = correct answer, 0 = error).

The physiological measurements were extracted from the signals recorded during the 2 minutes videos by means of the Acknowledge software ver. 4.2 (BIOPAC Systems, Inc.). The Skin Conductance Signal (SCL, orthosympathetic system measure) was low band filtered at 40 Hz, and the SCL values were obtained by subtracting the minimum value from the maximum value for each video. In order to have a physiological signal which was representative of the parasympathetic system, we computed the natural logarithm of the Respiratory Sinus Arrhythmia (RSA) from the pulse pressure wave and respiration signals. RSA gives a measure of the vagal tone as it reflects any variations in heart rate during inspiration and expiration^41^.

### Data analysis

Data analyses were conducted using Bayesian statistics. We used a Bayesian approach since this prevented us from incurring p-value related controversies with the aim of mitigating any problems due to the lack of replicability of the results^42–45^. Bayesian multilevel hierarchical models were used in all of the analyses^46,47^ (for an example in the frequentist context see^48^). This was done in order to take into account any within-subjects random variability that might have a confounding impact on the fixed, experimentally manipulated factors which address statistical population effects. In addition, since some of the participants took part in both experiments, data from both experiments were analysed in a single analysis, and the random intercepts and slopes were computed for each participant. As a result, individual deviations from the fixed factors for those participants who performed in both sessions were taken into account. For each analysis and for each factor, we computed the Bayes Factors (BF_10_^49^) via the Kuo and Mallick algorithm^50^. In order to interpret the results, we followed the seminal recommendations by Raftery^51^ who reports a BF_10_ within [3 ÷ 20) as an index of *positive evidence*, a BF_10_within [20 ÷ 150) of *strong evidence*, and greater probabilities as indexes of *very strong evidence* in favour of the alternative hypothesis (i.e. the presence of differences between the levels of the factor or interaction). Conversely, a BF_10_ lower than 0.0067, within (0.0067÷ 0.05], and within (0.05 ÷ 0.33] are, respectively, indicators of very strong, strong and positive evidence in favour of the null hypothesis (i.e. the absence of a difference between the levels of the factor or the degree of interaction)(Lodewyckx *et al*., 2011). Finally, a BF_10_ within (0.33÷ 1) and within (1 ÷ 3) are classified as weak support for the alternative or null hypothesis, respectively, while a BF_10_ = 1 constitutes no support for any hypothesis^52^. For each analysis, we used 5 Monte Carlo Markov Chains with 15000 iterations each, 2000 burn-in steps and 2000 adaptation steps.

Further details of the statistical procedure are shown in the Supplementary Materials (SM1).

Data analyses using SCL and RSA as dependent variables are reported in the Supplementary Materials 2 (SM2).

All the results are reported in the main text. However, only positive, strong or very strong evidence is analysed in depth (by means of a post-hoc analysis) and discussed.

#### Data analysis of the representation of PPS around the feet

In order to take into account any variances in the behavioural data as a whole, we used Bayesian linear models. In particular, we used specific models which took into account the variances in the whole data set when computing the CCEs (see more details in the Supplementary Materials - Table SM1).

The BF_10_ of the principal effects and the interactions between them were calculated. In the presence of PPS representation, Unilateral CCEs are expected to be greater than Bilateral CCEs. Thus, the presence of a difference between Unilateral and Bilateral CCEs constitutes evidence supporting an alternative hypothesis involving the Laterality factor and/or its interactions with other main effects.

Whenever Laterality or its interactions with other main effects provided evidence supporting the alternative hypothesis, the Unilateral – Bilateral contrast was further tested. These Post-hoc tests were repeated using a Bayesian CCE-linear model (Table SM1).

We then computed the estimates of CCEs (see the model in Table SM2). Estimates are reported as the Mode (Mo) and the 99% Highest Posterior Density Interval (HPDI). The 99% HPDI is the Bayesian analogue of the frequentist Confidence Interval. More precisely, this is the narrowest interval of the underlined curve of which contains 99% of the area^53^. In all cases, the $\hat{R}$ values were computed. $\hat{R}$ values within [1 ÷ 1.1) indicate a good convergence between the chains of the posterior distribution.

#### Data analysis of verbal feedback concerning sensations of movement

The verbal feedback given by the participants regarding their perception of sensations of movement was analysed in terms of accuracy (i.e. correct answers to the direct question “Were your legs moved?”). This was done means of Hierarchical Bayesian Binomial models (see Table SM3). We also analysed these data to test whether the correct answers really were correct (i.e. there was a probability of them being correct of 85%), and establish that there were no differences between these responses and random responses (i.e. the null hypothesis, with a probability of 50% of a correct answer). This model is reported in Table SM4.

#### Covariance analyses between the PPS around the feet and clinical, physiological and interoceptive sensibility scores

In order to investigate potential relationships between the representation of PPS around the feet and physiological responses (SCL and RSA) and BPQ scores (the index of interoceptive sensibility^34^), two covariance analyses were carried out taking into account only the Unilateral data. Furthermore, for the CP and IP participants, we covaried the PPS representation around the feet with the AIS and NLI data which were converted into numerical form (i.e. AIS: A = 1, …, E = 5; NLI: C1 = 1, …, S5 = 30 – coccygeal lesions were not included). The models shown in Table SM1 and in Table SM2 were used for all of the analyses of the estimates.

## Results

### Baseline comparison

As a first step, the CCEs in the Baseline and Follow-Up conditions were analysed in order to test the null hypothesis (i.e. the absence of differences) via a CCE linear model (Table SM1). In this analysis, the fixed factors were: Laterality (Bilateral, Unilateral), Condition (Baseline, Follow-Up) and the interaction between these. As all of these factors are within-subject, we also used them as random effects, grouped by participant. In cases where there was a difference in the PPS representation between Baseline and Follow-up, one would expect the Laterality X Condition interaction to have a BF_10_ of at least 3^49^. However, the results showed that the null hypothesis can be accepted (BF_10_ = 0.06), namely the PPS representation in the Baseline and Follow-Up conditions were not different. As a result, the Baseline and Follow-Up blocks were united in a single “No Stimulation” condition which took into account average learning and fatigue effects. This represents the only control condition in the following analyses.

### Representation of PPS around the feet

We used the CCE linear model in Table SM1 to test the full experimental design. The fixed effects were Group (CP, IP, C), Condition (No Stimulation, Vision: Mobilisation – Motion: Mobilisation, Vision: Mobilisation – Motion: No Mobilisation, Vision: No Mobilisation – Motion: Mobilisation, Vision: No Mobilisation – Motion: No Mobilisation), Laterality (Bilateral, Unilateral) and the interactions between them. The random effects were the within-subject factors (Laterality, Condition and the interaction between them) and the numerical order of the blocks to consider the effects of attention, fatigue and learning. The order of the blocks was converted into z-scores to obtain a zero-centred continuous variable to avoid spurious results. The random effects were varying slopes grouped by participant.

Strong effects for Laterality (BF_10_ = 86.71), positive effects for Group (BF_10_ = 3.56), and a very strong 3-way interaction between Group:Condition:Laterality (BF_10_ > 150) were found, suggesting the presence of differences in PPS representation depending on Group and Condition. However, the Group:Condition interaction showed a null effect (BF_10_ = 0.02), which indicates that there is not a general difference in RTs between Groups and Conditions, and the previous 3-way interaction is an effect produced by the stimuli within or outside the PPS. All of the remaining results showed only weak support for the alternative or null hypotheses [BF_10_ within (0.55÷1.08), but never = 1] and were thus not considered in the subsequent analyses.

Post-hoc analyses of the Group:Condition:Laterality interaction confirmed the presence of a PPS representation around the feet in the C group in the “No Stimulation” condition (BF_10_ = 7.85) and for the conditions where visual and motor stimulation were congruent [i.e. “Vision: Mobilisation – Motion: Mobilisation” (BF_10_ = 54.56) and the “Vision: No Mobilisation – Motion: No Mobilisation” (BF_10_ = 124)]. In contrast, in the “Vision: No Mobilisation - Motion: Mobilisation” condition only weak support for the alternative hypothesis was found (BF_10_ = 1.64) and in the “Vision: Mobilisation - Motion: No Mobilisation” condition there was positive evidence supporting the null hypothesis, namely the absence of a PPS representation (BF_10_ = 0.08).

In the case of the CP group, there was only evidence of a PPS representation around the feet in the conditions where the passive movement was actually administered. In fact, PPS was present in the “Vision: Mobilisation - Motion: Mobilisation” (BF_10_ = 15.13) and “Vision: No Mobilisation – Motion: Mobilisation” (BF_10_ = 20.28) conditions, while in the “No Stimulation” (BF_10_ = 0.04), “Vision: Mobilisation - Motion: No Mobilisation” (BF_10_ = 0.15) and “Vision: No Mobilisation -Motion: No Mobilisation” (BF_10_ = 0.329) conditions there was positive or strong evidence supporting the null hypothesis, suggesting a lack of PPS representation.

**Figure 2:**
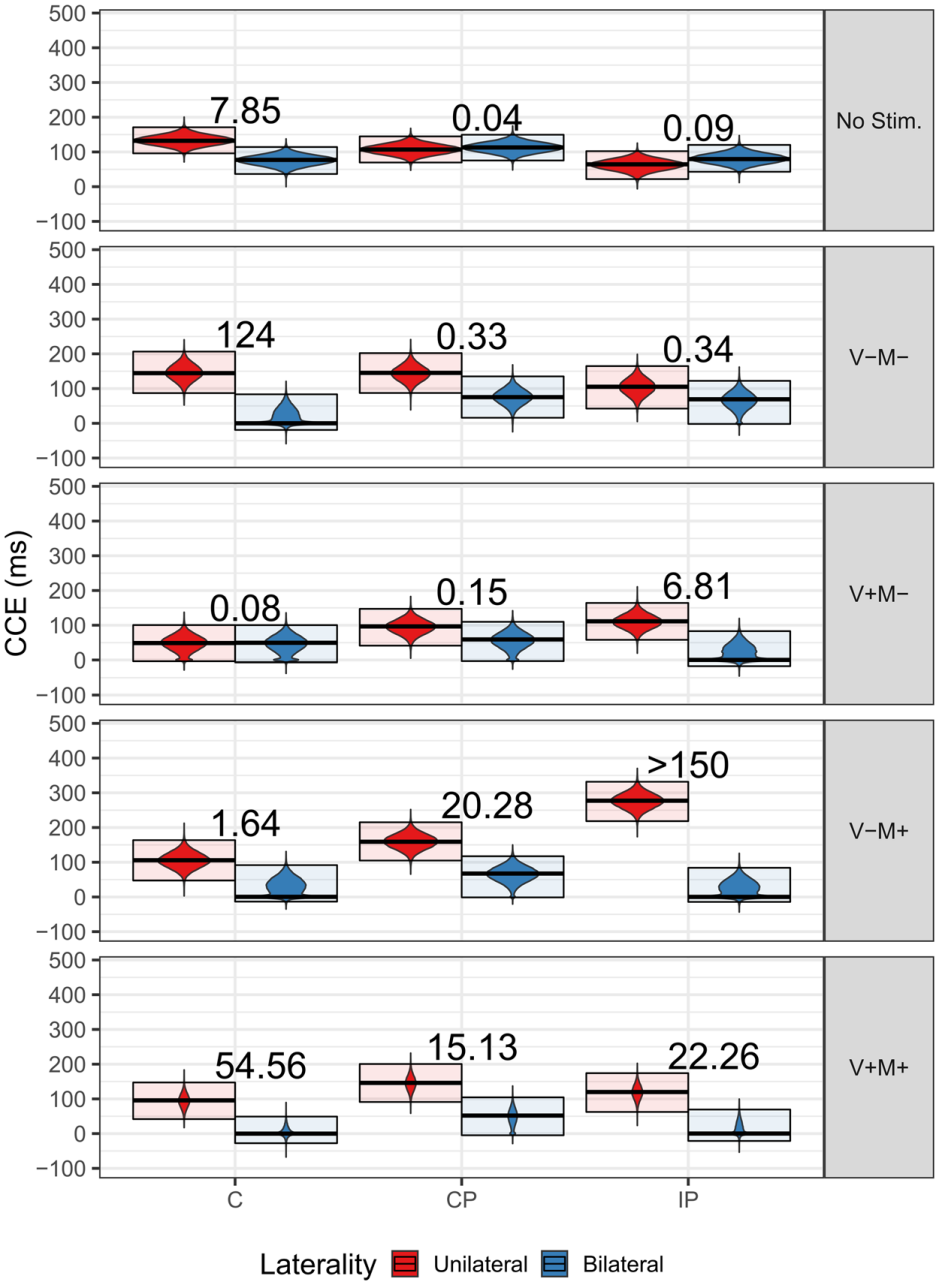
Posterior distributions of CCEs from the Bayesian CCE-linear model. The distributions are represented by violin plots. The darker line in the middle of the box is the median, and the upper and lower boundaries of the box represent the first and the third quartile. The curves are probability density curves represented along the y-axis instead of the x-axis, plotted on each side. The numbers are the BF_10_ for Unilateral v. Bilateral differences. No Stim. = No Stimulation; V-M- = Visual: No Mobilisation – Motion: No Mobilisation; V + M- = Visual: Mobilisation – Motion: No Mobilisation; V-M + = Visual: No Mobilisation – Motion: Mobilisation; V + M + = Visual: Mobilisation – Motion: Mobilisation; C = Control Group; CP = Complete Paraplegics Group; IP = Incomplete Paraplegics Group.

The results for the IP group showed a similar pattern to the CP group, with strong evidence of the presence of a PPS representation around the feet in the “Vision: Mobilisation – Motion: Mobilisation” (BF_10_ = 22.26) and “Vision: No Mobilisation – Motion: Mobilisation” (BF_10_ > 150) conditions. Nevertheless, in this group, PPS was also recorded in the “Vision: Mobilisation – Motion: No Mobilisation” condition (BF_10_ = 6.81), while in the “No Stimulation” condition there was strong evidence supporting the null hypothesis (BF_10_ = 0.09, absence of PPS representation), and in the “Vision: No Mobilisation – Motion: No Mobilisation” condition there was only weak evidence supporting the null hypothesis (BF_10_ = 0.34). See Fig. 2 and Table 2 for the estimates of posterior distributions.

**Table 2:** Estimates from the Bayesian CCE-linear model in milliseconds. In each cell are the mode and, within square brackets, 99% of the Highest Posterior Density Interval.

Condition	Laterality	Group		
		C	CP	IP
*No Stimulation*	*Bilateral*	273.32 [232.69–310.28]	309.14 [271.58–345.68]	275.6 [239.16–316.42]
	*Unilateral*	328.29 [292.13–367.13]	303.07 [265.74–340.94]	260.51 [217.98–298.42]
*Visual: Mobilisation* *Motion: Mobilisation*	*Bilateral*	196.08 [168.56–245.16]	248.1 [191.37–300.55]	196.32 [175.08–265.46]
	*Unilateral*	291.79 [237.94–343.37]	342.21 [287.31–396.62]	315.95 [258.54–370.24]
*Visual: Immobile* *Motion: Mobilisation*	*Bilateral*	196.51 [182.89–287.85]	263.57 [194.94–313.43]	196.4 [182–280.18]
	*Unilateral*	301.88 [243.6–359.72]	355.15 [301.07–411.21]	473.4 [414.51–527.9]
*Visual: Mobilisation* *Motion: Immobile*	*Bilateral*	245.66 [189.96–296.47]	255.24 [192.89–306.02]	196.28 [179.25–279.12]
	*Unilateral*	244.75 [192.47–296.67]	292.75 [237.43–343.33]	307.4 [254.17–360.31]
*Visual: Immobile* *Motion: Immobile*	*Bilateral*	196.22 [177.04–279.66]	271.55 [212.15–331.22]	265.26 [194.26–318.46]
	*Unilateral*	340.63 [283.13–402.73]	341.4 [283.51–398.25]	301.48 [238.57–360.92]

### Movement verbal feedback

In this analysis, the fixed effects were Group (C, IP, CP) and Condition (Vision: Mobilisation – Motion: Mobilisation, Vision: Mobilisation - Motion: No Mobilisation, Vision: No Mobilisation - Motion: Mobilisation, Vision: No Mobilisation - Motion: No Mobilisation), and the interaction between them. The within-subject Condition factor was also used as random slopes which was grouped among participants.

This analysis showed very strong evidence supporting the alternative hypothesis for the Group:Condition (BF_10_ > 150), showing that there were differences in the accuracy of Movement Verbal Feedbacks between groups and conditions, but only weak, positive evidence supporting the null hypothesis for the Group (BF_10_ = 0.02, no differences among groups) and Condition (BF_10_ = 0.34, inconclusive evidence towards the lack of differences) factors.

An interaction emerged from the varying responses of the groups in the various conditions. The C group performed almost perfectly in all of the conditions. Indeed, they always recognised the presence or absence of movement with only one participant giving a wrong answer (out of 42 trials) in the “Vision: No Mobilisation - Motion: No Mobilisation” condition.

The CP group performed perfectly in the “Vision: No Mobilisation - Motion: Mobilisation” condition (42/42), but made mistakes in the “Vision: Mobilisation - Motion: Mobilisation” (34/42), “Vision: Mobilisation - Motion: No Mobilisation” (37/42), and “Vision: No Mobilisation - Motion: No Mobilisation” (38/42) conditions. In fact, while the comparison between the “Vision: No Mobilisation - Motion: No Mobilisation”, “Vision: Mobilisation - Motion: Mobilisation” and Vision: Mobilisation - Motion: No Mobilisation” conditions revealed strong, positive evidence supporting the null hypothesis (namely the absence of a difference between conditions), the comparisons with the “Vision: No Mobilisation - Motion: Mobilisation” condition all showed strong or positive evidence supporting the alternative hypothesis (presence of differences between conditions). Thus, in the condition where CP participants saw immobile legs but their own legs were actually being moved, they were better at detecting movement.

**Figure 3:**
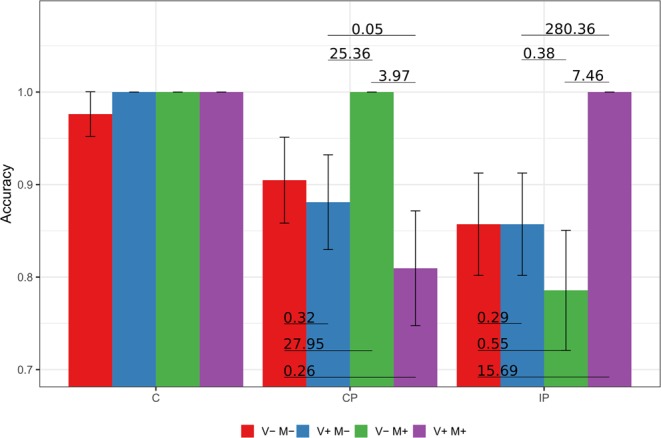
Proportion and standard error of the accuracy in the Movement Verbal Feedback. C = Control Group; CP = Complete Paraplegics Group; IP = Incomplete Paraplegics Group. The numbers on the lines connecting the bars are the BF_10_.

Conversely, the IP group performed perfectly in the “Vision: Mobilisation - Motion: Mobilisation” condition (42/42), while they made mistakes in the “Vision: No Mobilisation - Motion: No Mobilisation” (36/42) “Vision: Mobilisation - Motion: No Mobilisation” (36/42) and “Vision: No Mobilisation - Motion: Mobilisation” (33/42) conditions. Comparisons between the “Vision: Mobilisation - Motion: Mobilisation” and “Vision: Mobilisation - Motion: No Mobilisation” conditions (and consequently also “Vision: No Mobilisation - Motion: No Mobilisation”), and between the “Vision: Mobilisation - Motion: Mobilisation” and “Vision: No Mobilisation - Motion: Mobilisation” conditions revealed positive, strong evidence supporting the alternative hypothesis (“therefore the performances in all these conditions were different). The comparison between their performance in the “Vision: Mobilisation - Motion: No Mobilisation” and in “Vision: No Mobilisation - Motion: Mobilisation” conditions (and, consequently, also in “Vision: No Mobilisation - Motion: No Mobilisation” and “Vision: No Mobilisation - Motion: Mobilisation”) showed weak evidence supporting the null hypothesis (therefore an inconclusive tendency towards a lack of differences between the conditions). See Fig. 3 for a graph representing these results.

Lastly, we checked in order to ensure that the performances were better than random guesses using the model in Table SM4 for each group and condition. This was confirmed by the presence of very strong evidence supporting the alternative hypothesis in all the conditions and groups (all BF10 > 99).

### Covariations of physiological responses with the representation of PPS around the feet

The analysis of the relationship between the CCEs and the physiological data involved Group (C, IP, CP), Condition (No Stimulation, Vision: Mobilisation - Motion: Mobilisation, Vision: Mobilisation - Motion: No Mobilisation, Vision: No Mobilisation - Motion: Mobilisation, Vision: No Mobilisation - Motion: No Mobilisation), the Skin Conductance Level (SCL) and the Respiratory Sinus Arrhythmia (RSA) covariates and their interactions. The random factors considered were the same as those used in the behavioural analysis, that is, Condition and the number of blocks in z-scores, grouped for each participant. There was positive evidence supporting the alternative hypothesis in the interactions between Condition and SCL (BF_10_ = 4.07) and between Condition and RSA (BF_10_ = 8.13). The SCL covaried with the PPS representation as measured by CCEs in the “Vision: No Mobilisation - Motion: No Mobilisation_”_ condition (BF_10_ = 75.92), and this covariation was positive, with greater CCEs linked to higher SCL responses (Mo = 0.25; HPDI = −0.01, 0.44). In the other conditions, the BF_10_ were within [0.70 ÷ 1.44], showing only weak, negligible evidence. The RSA positively covaried with CCEs in the Vision: No Mobilisation - Motion: Mobilisation condition (BF_10_ = 17.18; Mo = 0.23; HPDI = −0.01, 0.37), that is, greater CCEs were linked to higher RSA responses (Mo = 0.23; HPDI = −0.01, 0.37). In all of the other conditions, BF_10_ was within [0.75 ÷ 1.5], showing weak, non-conclusive evidence.

Finally, positive evidence supporting the null hypothesis was found in the 3-way interaction between Group, Condition and SCL (BF_10_ = 0.24), between Group and RSA (BF_10_ = 0.27) and in the main effect Group (BF_10_ = 0.19).

### Covariations of interoceptive awareness with the representation of PPS around the feet

An evaluation of interoceptive awareness was made by analysing the Body Perception Questionnaire scores in relation to the representation of PPS around the feet following the same structure as that used in the previous analysis. There was very strong evidence supporting the alternative hypothesis in the Group:Condition:BPQ interaction (BF_10_ = 305.12). In the case of Condition (BF_10_ = 7.35_)_ and BPQ, there was positive evidence supporting the alternative hypothesis (BF_10_ = 3.06). In particular in the IP group, BPQ positively covaried with PPS representation (CCEs) in the “Vision: No Mobilisation - Motion: Mobilisation” (BF_10_ = 32.33_;_Mo = 1.21; HPDI = 0.61, 1.77) and “Vision: Mobilisation - Motion: No Mobilisation” conditions (BF_10_ = 3_;_ Mo = 1.26; HPDI = 0.36, 2.29). This suggests that, in cases of incomplete lesions, greater interoceptive sensibility is associated with a better representation of PPS in conditions where motor and visual stimuli are incongruent.

### Covariations of clinical scores with the representation of PPS around the feet

The clinical scores for AIS and NLI were covaried with the PPS representation around the feet by taking into consideration the same fixed and random effects as those used in the previous covariation analyses. Positive evidence supporting the alternative hypothesis was found only in the NLI covariate (BF_10_ = 4.88) where cases of a lower degree of lesion – less deficit were associated with a better PPS representation around the feet (Mo = 0.42; HPDI = 0.33, 0.48). The other factors were not correlated with PPS representation, (BF_10_ were within [0.64 ÷ 2.03]).

## Discussion

In the present study, immersive virtual reality technology was used in order to investigate the impact of SCI on the relationship between visual and bodily information in the modulation of PPS. In two experimental sessions, participants observed virtual reality videos in which a pair of lower limbs seen from a first-person perspective were passively moved or remained immobile. Simultaneously, the participants’ own legs were either being manually moved or were not moved. Behavioural, physiological, and subjective reports were recorded. In spite of massive anaesthesia, the results indicate that bodily information plays an important role in the representation of PPS in SCI patients. In fact, in the CP and IP participants, the presence of passive movement was sufficient to restore PPS representation regardless of the visual feedback (both in “Vision: Mobilisation - Motion: Mobilisation” and “Vision: No Mobilisation - Motion: Mobilisation”). Conversely, in the group of controls, the mismatch between visual and motor feedback disturbed the representation of PPS.

When explicitly asked, the CP participants were able to accurately identify (i.e. always better than random guessing) the presence of movement (even though it was not visible), suggesting that some residual perception of movement may persist even after complete SCI. Nevertheless, our results indicate that the processes underlying the recovery of PPS are, at least in part, implicit and probably linked to interoceptive functions that may be less affected by the lesion.

It has been suggested that CCE is modulated by attentional processes relating to response mapping competition^54,55^. Although we did not assess this aspect directly, the attentional component was kept constant in our experiments, as also confirmed by the absence of any differences in performance between baseline and follow-up. We can thus consider our measure of CCE as a specific index of PPS representation. We found a reduction in PPS associated with SCI-related deafferentation/deefferentation in both CP and IP participants. In fact, in the baseline and follow-up conditions (which did not include the virtual reality visual feedback or manual passive Mobilisation), only the C group showed a CCE (as an index of PPS representation). These data are in line with the observation made by Bassolino and colleagues^56^ that PPS is reduced as a result of a lack of ability to move. They also reinforce the data from our earlier study which provided evidence that paraplegic participants with complete lesions showed a reduction in PPS around their feet but not around their hands^21^. Interestingly, it seems that this is not true for upper-limb affordances. Indeed, in a study on 10 patients with heterogeneous SCIs, Sedda and colleagues^57^ did not find faster affordance judgements on the reachability of objects in peripersonal space, while in the control group they found the effect of PPS judgement.

### The effect of passive motion and multisensory incongruence on the representation of PPS around the feet

PPS is defined as the “space in which actions are performed”, and PPS representation is considered to be associated with action representation^58^ and with the actual possibility of moving. Recently, a broader definition proposes that “*PPS is a network of body-part-centered representations responsible for the actions toward, and in avoidance of, objects and other living entities, including people*.”^59^. In fact, PPS representations can be modulated by several top-down variables, such as social contexts^60^, defensive components^61,62^, anxiety levels^63,64^, body representation^29,65,66^ and non-defensive action capabilities^21,67,68^. This complexity suggests a multilevel network organisation^59,69^.

PPS is usually assessed by applying visual and tactile stimuli to the same body part. However, there is some evidence which indicates that the visual component of PPS is plays a stronger role in the detection of whether a stimulus is within or outside the PPS^22^. Most important for the aim of this study is the fact that separate PPS representations anchored to the hand, face, or trunk do in fact exist, but they are not independent of each other^70^.

The present study focuses on the relations between PPS and body representations.

The data collected confirm that lower lesions (i.e. fewer deficits) are associated with a greater representation of PPS around the feet in both the CP and IP participants, indicating that changes in the representation of space are directly related to sensory-motor deficits.

The results of this study also show that both active (as seen in other studies on healthy participants^28^) and passive movements can improve PPS representation. This is in line with an observation that, in upper limb amputees, the representation of PPS around the hands shrinks to the stump but can be enlarged by a wearable prosthesis^67^. However, until now it has not been totally clear whether this effect is due to (i) visual information about body shape, (ii) the possibility of actually performing movements using a prosthesis or (iii) an interaction between these two mechanisms. It was observed for the first time in an earlier study involving complete paraplegics^21^ that the plasticity of the PPS around the feet is mainly linked to the movement of the lower limbs (even passively) rather than to body shape. It was found that (probably latent) bodily information plays a role in modulating PPS representation. In the present study, the performance of the healthy controls suggests that consistency and congruency in the stimuli coming from different sensory modalities are needed in order for PPS representation to be preserved. In effect, our controls show an intact PPS representation in all of the conditions in which visual and motor information are congruent. However, visuo-motor incongruency induced a subjective mismatch between what was seen and felt that ultimately lead to an impairment of the lower-limb related PPS representation. In situations involving incongruent cross-modal stimulation, our cognitive system tends to integrate the information in a single percept^71,72^ that may lead to the subject in question interpreting the experience as “my legs are not really moving”. Another possibility is that the sight of one’s own legs being moved without concomitant proprioceptive feedback (and vice-versa) results in the perception of an impossible action. This might inhibit the representation of PPS as a consequence of the lack of activation of the networks which are usually involved in the internal representation of actions^73–75^. Since the same cortical networks are also activated in PPS representation (i.e. the intraparietal sulcus, lateral occipital complex and premotor cortex^76^), it is possible that observing an impossible action interacts with and inhibits PPS representation.

Crucially, this multisensory integration does not seem to help people who have suffered SCI, in particular when they are affected by a complete lesion. In effect, we observed that, in the CP group of participants in our study, visual stimulation was not enough to recover PPS representation, while the presence of actual passive mobilisation (even with the visual stimulation showing immobile legs in a resting position) led to the recovery of PPS. In fact, the CP participants recovered PPS representation in both the “Vision: Mobilisation - Motion: Mobilisation” and “Vision: No Mobilisation - Motion: Mobilisation” conditions. This represents an unexpected result as in cases of complete lesion, not only motor but also somatosensory pathways are completely interrupted, and no sensory information should reach the somatosensory cortex. However, the CP participants correctly reported the presence of movement in their subjective verbal responses, in particular in the “Vision: No Mobilisation - Motion: Mobilisation” condition. Moreover, during the informal interview after the experiment, these participants often said that after the injury, they had progressively learned to pay attention to new sensations (e.g. similar to chills) from the above-lesion body parts and from inside their body (interoception) that signalled their lower limbs were being moved. Thus, the improvement in PPS representation in this group in the conditions where movement was induced may be due to these above-lesion signals and their integration with higher level cognitive functions (i.e. embodiment of the legs seen in the first person, action representation and motor imagery).

Another possibility is that this recovery is an effect of so-called “discomplete lesions”^77^, namely, lesions which are complete from a functional and clinical point of view, but that maintain some distal forms of brain-body connections which the patient is unaware of.

In the present study, the paraplegics with incomplete lesions showed a recovery of the representation of PPS around their feet in all of the conditions where there was manual Mobilisation and/or a visual component involving movement (that is to say, in all conditions with the exception of the Vision: No Mobilisation - Motion: No Mobilisation condition). This is not surprising with regard to the Vision: No Mobilisation - Motion: Mobilisation and Vision: Mobilisation - Motion: Mobilisation blocks as these patients have residual somato-sensory functions.

The recovery of PPS in the Vision: Mobilisation - Motion: No Mobilisation condition is however more difficult to explain. Indeed, the IP group seemed to be sensitive to the incongruence between visual and motor signals, but in an opposite direction with respect to the group of controls, that is, PPS representation in incongruent blocks was reduced in Controls and improved in IP. We suggest that incongruent conditions had the effect of making the IP group shift their attention from the movement (which was absent) to bodily information. This may have led to an increased representation of their body in the space around them with indirect effects on PPS representation. In effect, only in these blocks was PPS representation recovery correlated positively with the degree of interoceptive awareness (as measured by means of the Body Perception Questionnaire).

### A potential contribution from interoception

Data regarding the changes in autonomic responses after SCI have been to date meagre. It has been found that autonomic parasympathetic control functions are not impaired by paraplegia^78,79^, while the orthosympathetic route is influenced by lesions higher than T5^78^. In contrast, disorders in autonomic responses have been reported after cervical lesions^80,81^. Our expectation was that, as a consequence of the lack of somatosensory input, there would be an increment in the sensitivity of the paraplegic participants to signals from inside their body which would lead to an increase in interoceptive responses. However, we did not find any difference in autonomic responses between the three groups. Furthermore, as part of a series of additional analyses, the SCI participants were divided by lesion level (<T5 or ≥T5) and completeness, but there were no differences in either RSA or SCL.

It is worth noting that the experiment was not designed to modulate SCL or RSA responses. Indeed, the stimuli depicted either a slow, smooth passive motion in the lower limbs, or no motion at all, accompanied by constant contact with the two experimenters’ hands on the participant’s thighs even though the faces of the experimenters were always out of sight (as shown in Fig. 1b). Thus, there were no “arousing” stimuli that could modulate SCL, or different “social” conditions that could stimulate RSA. Thus, the increase in SCL in the Vision: No Mobilisation - Motion: No Mobilisation condition might be due to a general effect of embodiment. In fact, it has been found that the orthosympathetic system is activated in healthy participants during motor imagery tasks when these are executed from a first-person perspective but not from a third-person perspective^82^. Furthermore, our results may be in keeping with reports of the activation of the parasympathetic system during passive movement (Vision: No Mobilisation - Motion: Mobilisation)^83^.

A potential role of interoception in PPS recovery also seems to be supported by the results from the questionnaire on interoceptive awareness (BQP) which positively covaried with PPS representation in the IP participants in the incongruent conditions. This suggests that SCI patients with a higher interoceptive awareness might also refer to this typology of information when faced with conflicting, discrepant visuo-motor stimuli.

The connection between PPS representation and interoception was previously observed in healthy participants by Ferri, Chiarelli, Merla, Gallese, & Costantini^84^ and Noel *et al*.^85^. After 15 minutes of audio-visual deprivation, PPS representation becomes ill-defined. Although the participants’ responses in interoceptive accuracy were different (i.e. some indicating improvements and some worsening), the study found a correlation between PPS representation and the modulation of interoceptive accuracy^85^. Our results are consistent with these data indicating that individuals with better interoception show greater modulations of PPS representations.

### Somato-topographic cognitive modifications in SCI

Cognitive studies on SCI participants have demonstrated that body-brain disconnection (without brain lesions) leads to a somato-topographically specific reorganisation of several cognitive functions. Action representation alters after SCI. Although people with SCI report that they walk in their dreams^86^, there is evidence indicating that paraplegic patients may suffer from a dramatic reduction in motor imagery capacities^87–91^ and in their ability to discriminate biological motion (e.g. the direction of the ambulation of a point-light walker^4^). This is true even when they are aware of their motor impairment^92^. These deficits have been found to be topographically specific as they involve actions that regard the paralyzed body parts but not those which are habitually executed by the upper body parts^6,21^. It is also worth noting that while a recent study reported a deficit in learning new motor sequences^7^, other studies have shown that SCI patients acquire new post-lesional motor abilities and that this also leads to specific expertise in visual action discrimination^6,8^.

An interesting result from the present study is that not only action and body perception but also the representation of PPS were modified in the SCI participants according to topographically specific rules^21^. All in all, our study supports the notion that cognitive functions such as the representation of space are grounded on sensory-motor functions and bodily signals. In fact, if cognitive functions such as space representation was only based on high order, symbolic systems, no changes would be recorded after a lower level, spinal body-brain disconnection.

### Clinical implications of the cognitive effects of sensory-motor deficits

Widespread reorganisation of the brain has been documented after SCI^93,94^, with a probable role of deficit compensation and re-adaptation. Importantly, however, maladaptive plasticity leading to neuropathic pain and corporeal illusions^10,95,96^ has also been reported. These negative effects may impact on any approach to treatment based on EEG rhythms^97–99^. Thus, it is extremely important to ascertain whether there are any changes in cognitive functions after a sensory-motor deficit and to utilise tasks that can help people with SCI to maintain cognitive functions and contrast maladaptive plasticity^100,101^. Our results show that simple passive motion for only 2 minutes may lead to the functional recovery of space representation. Although this recovery did not last until the Follow-Up assessment, it may be the case that more focused, sustained protocols would result in more lasting outcomes. This would support the results of a number of previous studies which have indicated that motor rehabilitation has positive effects on cognition^102,103^.

## Conclusions

Overall, our results support the hypothesis that changes in cognitive functions follow conditions of deafferentation and deefferentation^10,104–106^. These modifications probably originate from the widespread neuroplastic modifications that occur after spinal cord lesions^93,107–111^ in neural regions underpinning both bodily representation and the mapping of peripersonal space^112^. These neuroplastic changes are mediated by multiple sources of information coming from the body. A perfect balance between these appears to be necessary to guarantee thecorrect representation of PPS.

Nevertheless, residual bodily information seems to assist the recovery of PPS in SCI. This supports the notion that bodily information is fundamentally important for the representation of PPS thus providing evidence supporting the notion of embodied PPS.

## Supplementary information

Supplementary Materials.

## Data Availability

Data and the JAGS scripts are available at: https://osf.io/mfa82/
