# Supplementary material for: Visuo-motor and interoceptive influences on peripersonal space representation following spinal cord injury
Source: Sci Rep. 2020 Mar 20;10:5162. doi: 10.1038/s41598-020-62080-1 (PMC7083926; doi:10.1038/s41598-020-62080-1)
Supplement: Supplementary file 1 — Supplementary Materials. [file 41598_2020_62080_MOESM1_ESM.docx]

# Supplementary Materials of: Visuo-motor and interoceptive influences on peripersonal space representation following spinal cord injury.

Authors: *Scandola M., Aglioti S.M., Lazzeri G., Avesani R., Ionta S. and Moro V.*

## SM1. Bayesian models:

Bayes Factors are ‘‘*the standard Bayesian solution to the hypothesis testing and model selection problems*’’ (Lewis and Raftery, 1997 p.648). We computed probability values for each factor that are equivalent to Bayes Factors via a transdimensional Markov Chain Montecarlo known as the Product Space Method (PSM, Carlin and Chib, 1995; Lodewyckx et al., 2011).

PSMs use a hyperprior model index (here identified with *ι*), which measures the proportion of times that each factor is used to account for the observed data. These proportions are indicated by *P(ι=1|D)*, with *ι* being the index of the factor, and D being the data. The *P(ι=1|D)* values are ranged from 0 to 1. Bayes Factors are then computed as follows: BF_10_ = *P(ι=1|D)/ P(ι=0|D).*

Through this methodology we compared, for each factor, the alternative hypothesis (i.e., the presence of that factor is necessary to explain the data distribution) with the null hypothesis (i.e. the presence of that factor does not explain the data distribution).

Here, all the results will be reported, but only positive, strong or very strong evidence will be further analysed and discussed. All the results show reliability with $\hat{R}$ values within [1 ÷ 1.1) (Gelman and Rubin, 1992; also known as the Gelman’s Diagonal, Brooks and Gelman, 1998).

These analyses have been computed within the R programming language for statistical analyses (R Core Team, 2017), using the jagsUI (Kellner, 2017) package to connect it to JAGS (Plummer, 2015). This is a GNU software used to perform Markov Chain Montecarlo simulations via the Gibbs sampling algorithm (Geman and Geman, 1984). For each model the chains were adapted with 2,000 iterations, then the chains were iterated 15,000 times, with 2,000 burn-in iterations. For each model we sampled from 5 chains, for a total of 75,000 iterations with 12,000 burn-in iterations.

All the Bayesian models are described in three parts: (i) Likelihood, which computes the data distribution parameters from the dependent variables; (ii) Priors, which computes the prior parameters used by the Likelihood (the coefficients from the fixed and random factors, and the ι indexes) from the non-informative prior distributions; and (iii) Hyperpriors, which computes the hyperparameters that are then used from the prior distributions.

### Table SM1. CCE-Linear models with PSM.

These models are conceived to take into account for the whole variability of the data for the Crossmodal Congruency Effect (CCE) The CCE is defined as the difference between the performance in *Incongruent* and *Congruent* trials, and is typically computed as difference between the means of the two series of values (Pavani et al., 2000; Maravita et al., 2002). This leads to the loss of data variability, and to the impossibility to estimate the coefficients of the model (βs) exploiting the full possibilities of linear multilevel models (Pinheiro and Bates, 2000; Gelman and Hill, 2006). In our analysis, to overcome these problems, within the same hierarchical Bayesian model we estimate coefficients for two linear models: in the first one we take into account the dependent variables of only *Congruent* trials (obtaining the β_Congruent_ values), while in the second one we take into account only the dependent variables for *Incongruent* trials (obtaining the β_Incongruent_ values). Then, the coefficients for the CCE (β_CCE_) are given by the differences between the β_Incongruent_ and β_Congruent_ coefficients.

The PSM is given by the estimation of ι index (that can take 0 or 1 values) for each factor, which give us the probability of that factor to be used to explain data variability.

“NR” is the number of the random coefficients, “NB” is the number of the fixed effects, “b” is the index for parameters that are connected with fixed effects, “r” is the index for parameter that are connected with random effects, “f” is the index for the factors and their interactions, “i” is the index for the dependent variable. “X” represents the matrix for fixed effects, “R” the matrix for random effects, “P” the matrix for the correlations among random coefficients, “S” the variance-covariance matrix among random coefficients. The “β” are the coefficients for the fixed effects, with parameters “µ” (mean) and “σ” (standard deviation). The “u” are the coefficients for the random effects, with parameters “ϖ” (mean) and “ς” (standard deviation). The “ϑ” coefficient is the variance for the likelihood. The “shape” and “rate” parameters for the gamma distributions, used as hyperpriors for the “σ” and “ϑ” coefficients, and they are equal to shape = SD(y)/2 and rate = 2SD(y) (Kruschke, 2014 p.561).

| ***Hyperpriors:***  For(b in 1:Number of Fixed Coefficients) 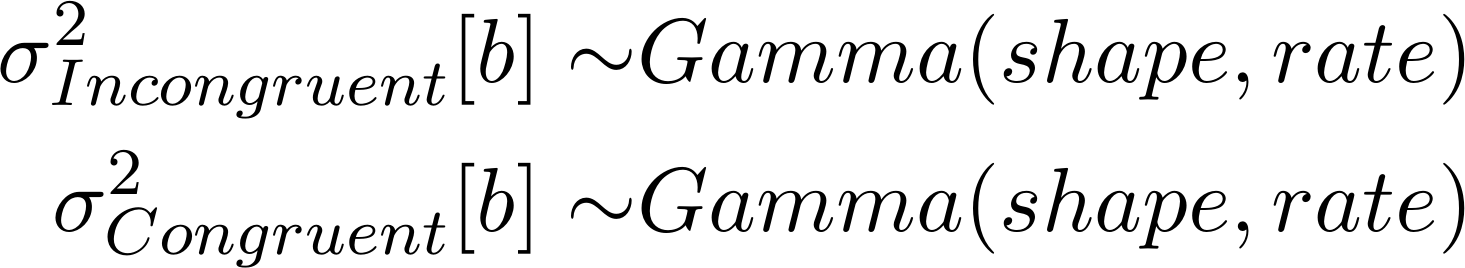 For(r in 1:Number of Random Coefficients) 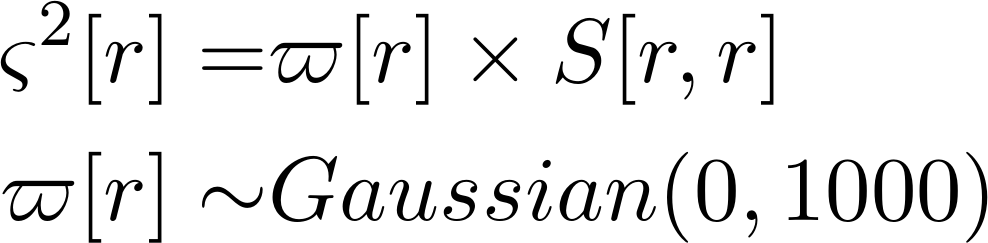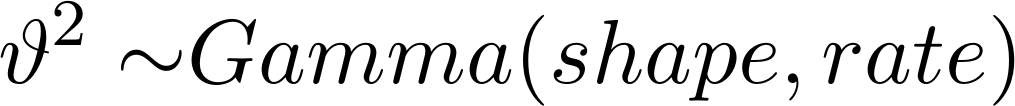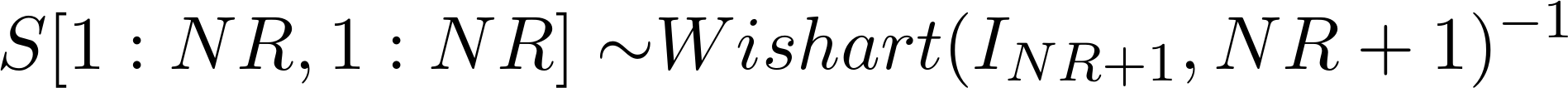 | The hyperprior distributions are non-informative (therefore no prior assumptions can be made). We determine the variance for the priors for the fixed coefficients (σ^2^_Congruent_ and σ^2^_Incongruent_), the mean and variance parameters for the random coefficients ($\varpi$and $\varsigma^{2}$), the common variance of the model (ϑ^2^) and the variance-covariance matrix for the random coefficients (S).  The fixed coefficients are the coefficients used to take into account the fixed factors, namely the experimentally manipulated factors. The random coefficients are the coefficients used to take into account the random variation that is not experimentally manipulated, such as the individual ability, learning effects or fatigue effects. | | |
| --- | --- | --- | --- |
| ***Priors:*** 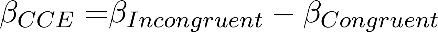 For(b in 1:Number of Fixed Coefficients) 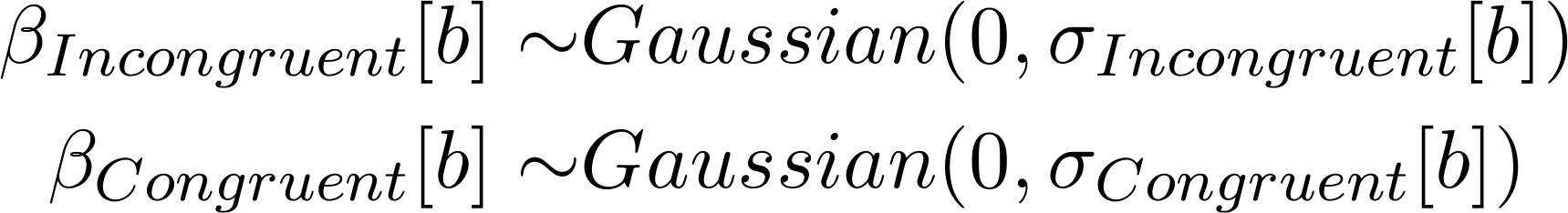 For(f in 1: Number of Factors) 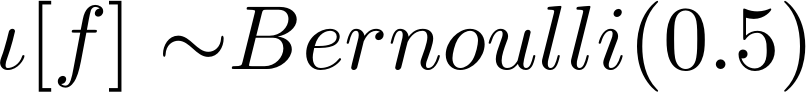 For(r in 1:Number of Random Coefficients) 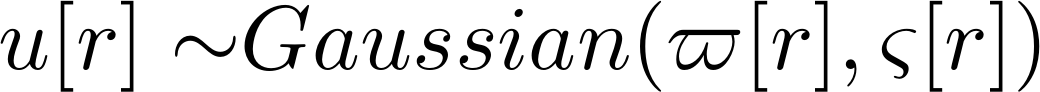 For(r1 in 1:Number of Random Coefficients)  For(r2 in 1:Number of Random Coefficients) 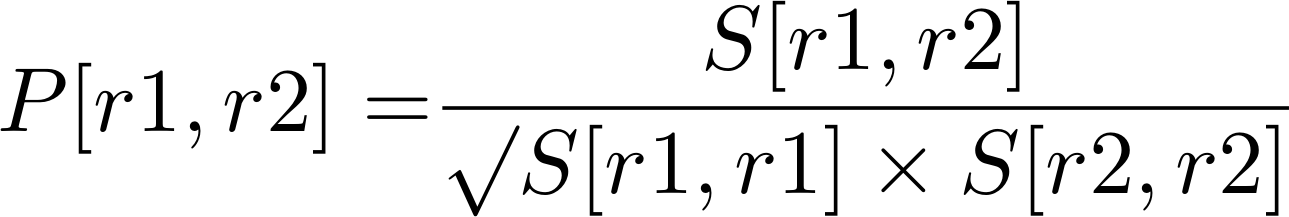 | | The prior distributions for the fixed, random and ι coefficients of the model.  The fixed effects coefficients (β_Congruent_ and β_Incongruent_) are determined by non-informative distributions. In particular, β_CCE_ = β_Incongruent -_ β_Congruent._  The ι index may take values within the [0÷1] range for all the factors from a non-informative distribution. This index is then adapted to take into account all the coefficients for each factor. When ι=1 in a single iteration, the factor is used to account for the data in that iteration.  Also, the random coefficients (*u*) are determined via non-informative distributions, whose parameters are the hyperprior distributions of *ϖ* and *ς*.  Here we also determine the correlation matrix among random coefficients (P), by using the hyperprior distribution of the variance-covariance matrix S. | |
| ***Likelihood:***  For(i in 1:Number of Observations) 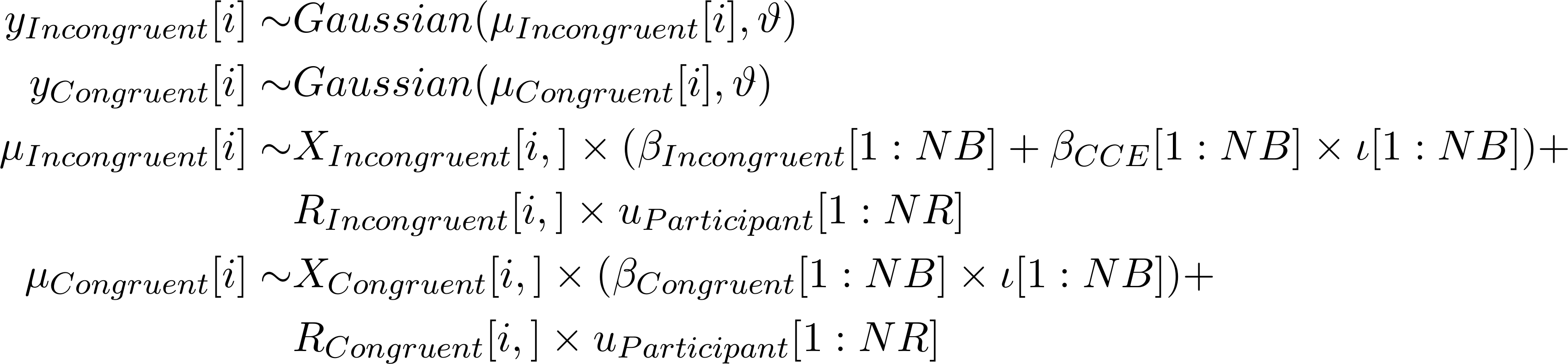 | | | The likelihood distributions of the dependent variables for the Congruent (y_Congruent_) and Incongruent (y_Incongruent_) trials are distributed according to Gaussian distributions, with mean µ_Congruent/Incongruent_ and standard deviation ϑ.  µ_Congruent/Incongruent_ are given by the linear combination of Fixed and Random coefficients. The fixed coefficients for the Incongruent trials are given by the sum of β_Congruent_ and β_CCE_, therefore β_CCE_ are the coefficients related to the peripersonal space representation.  The ι distribution can force to zero the β_CCE_ coefficients of a single factor, assuming the value of zero (therefore no peripersonal space representation). It is worth noting that β_Incongruent =_ β_Congruent +_ β_CCE._ |

### Table SM2. CCE-Linear models for estimates.

These models differ from the previous ones because there is not ι distribution. It is used to obtain precise β_CCE_ estimates from non-informative priors.

Description as in Table SM1 (except for the part regarding PSM).

| ***Hyperpriors:***  For(b in 1:Number of Fixed Coefficients) 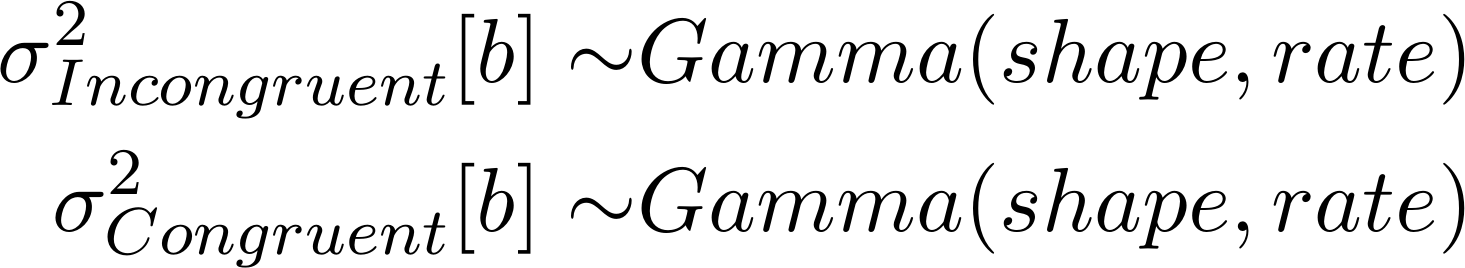 For(r in 1:Number of Random Coefficients) 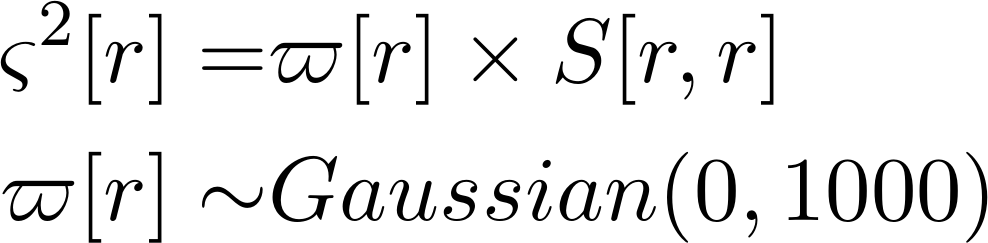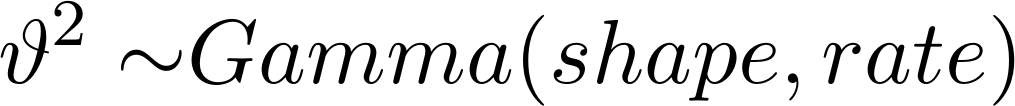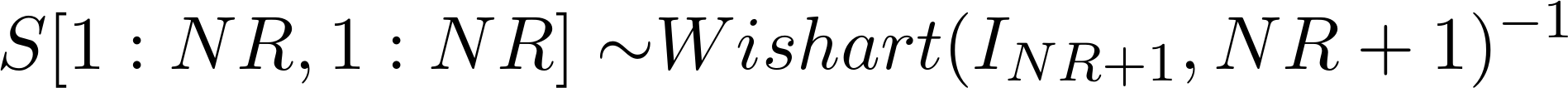 |  |
| --- | --- |
| ***Priors:*** 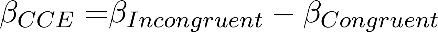 For(b in 1:Number of Fixed Coefficients) 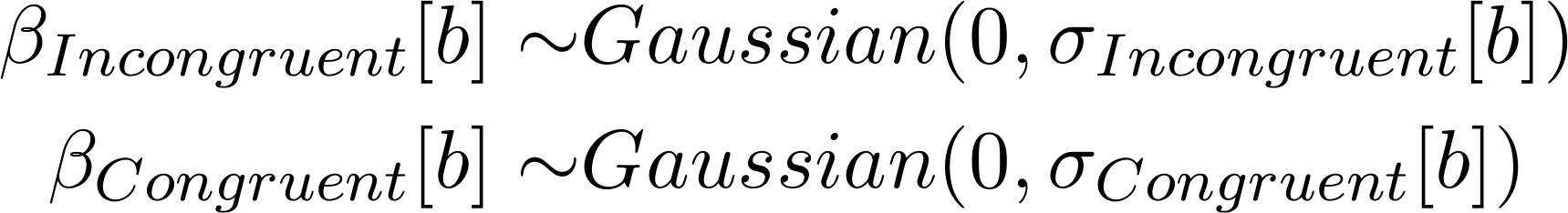 For(r in 1:Number of Random Coefficients) 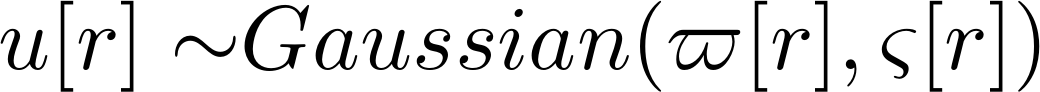 For(r1 in 1:Number of Random Coefficients)  For(r2 in 1:Number of Random Coefficients) 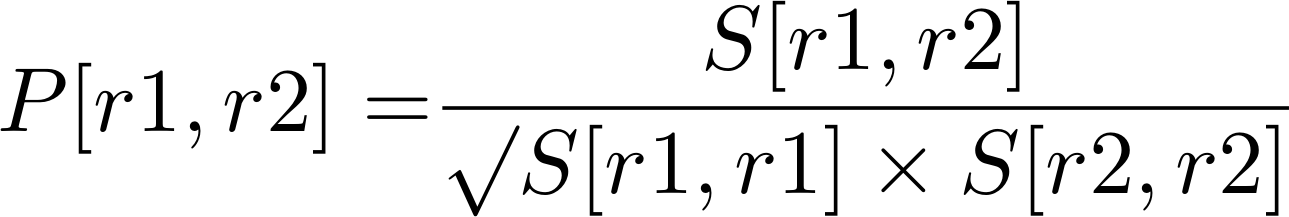 |  |
| ***Likelihood:***  For(i in 1:Number of Observations) 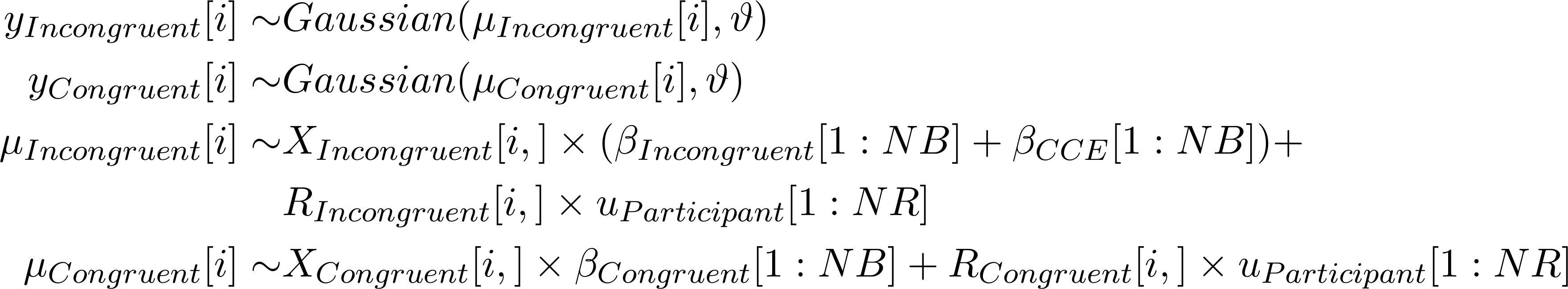 |  |

### Table SM3. Binomial models with PSM.

These models are used to study the Movement Verbal Feedbacks (MVP), which are the accuracies at the direct question “has been moved your leg?”. These data are binomial, for this reason we used Bayesian multilevel models for binomial data (Gelman and Hill, 2006; Kruschke, 2014).

The PSM is given by the estimation of ι index (that can take 0 or 1 values) for each factor, which give us the probability of that factor to be used to explain the data variability.

“NR” is the number of the random coefficients, “NB” is the number of the fixed effects, “b” is the index for parameters that are connected with fixed effects, “r” is the index for parameters that are connected with random effects, “f” is the index for the factors and their interactions, “i” is the index for the dependent variable. “X” represents the matrix for fixed effects, “R” the matrix for random effects, “P” the matrix for the correlations among random coefficients, “S” the variance-covariance matrix among random coefficients. The “β” are the coefficients for the fixed effects, with parameters “µ” (mean) and “σ” (standard deviation). The “u” are the coefficients for the random effects, with parameters “ϖ” (mean) and “ς” (standard deviation). The “ϑ” coefficient is the variance for the likelihood. The “shape” and “rate” parameters for the gamma distributions, used as hyperpriors for the “σ” and “ϑ” coefficients, and they are equal to shape = 1 and rate = 0.01. “θ” is the logit version of “µ” (namely the probability of getting a correct answer), and “N” is the total number of trials in that condition, group and subject.

| ***Hyperpriors:***  For(b in 1:Number of Fixed Coefficients) 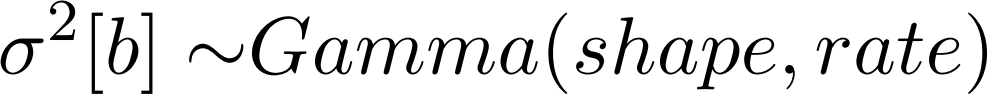 For(r in 1:Number of Random Coefficients) 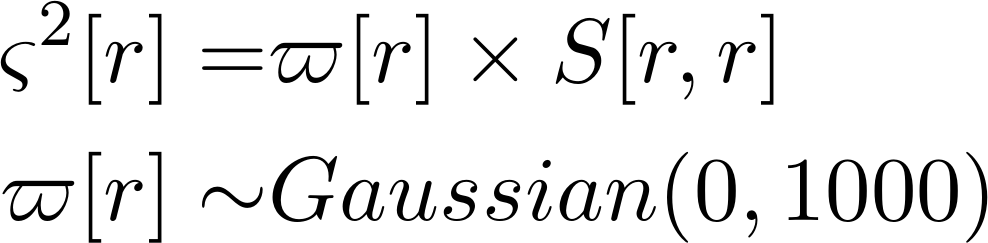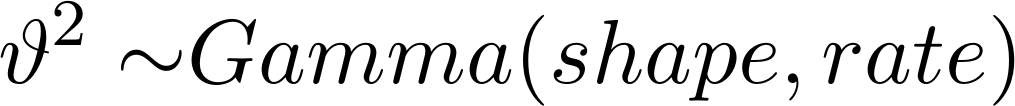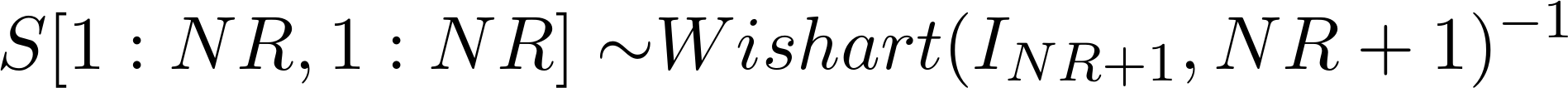 | The hyperprior distributions are non-informative (therefore no prior assumptions can be made). We determine the variance for the priors for the fixed coefficients (σ^2^), the mean and variance parameters for the random coefficients ($\varpi$and $\varsigma^{2}$), the common variance of the model (ϑ^2^) and the variance-covariance matrix for the random coefficients (S).  The fixed coefficients are the coefficients used to take into account the fixed factors, namely the experimentally manipulated factors. The random coefficients are the coefficients used to take into account the random variation that is not experimentally manipulated, such as the individual ability, learning effects or fatigue effects. | |
| --- | --- | --- |
| ***Priors:***  For(b in 1:Number of Fixed Coefficients) 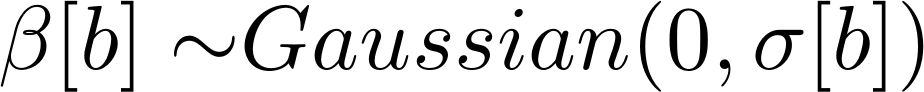 For(f in 1: Number of Factors) 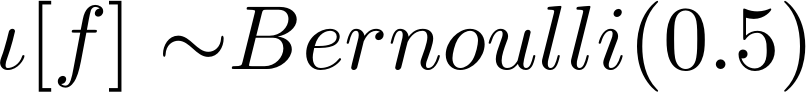 For(r in 1:Number of Random Coefficients) 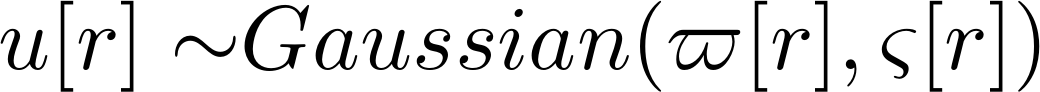 For(r1 in 1:Number of Random Coefficients)  For(r2 in 1:Number of Random Coefficients) 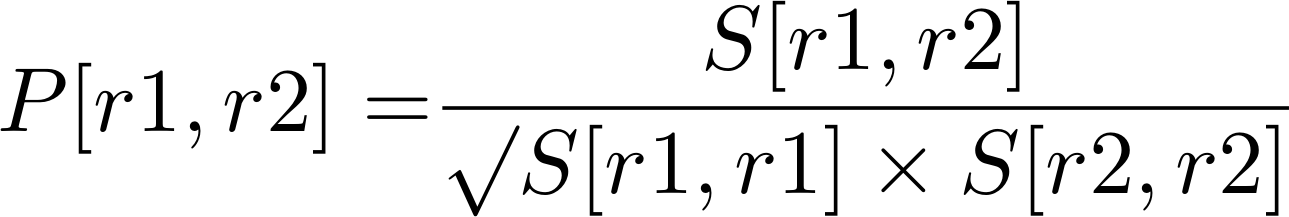 | | The prior distributions for the fixed, random and ι coefficients of the model.  The fixed effects coefficients (β) are determined by non-informative distributions.  The ι index may take values within the [0÷1] range for all the factors from a non-informative distribution. This index is then adapted to take into account all the coefficients for each factor. When ι=1 in a single iteration, the factor is used for accounting the data in that iteration.  Also, the random coefficients (*u*) are determined via non-informative distributions, whose parameters are the hyperprior distributions of *ϖ* and *ς*.  Here we also determine the correlation matrix among random coefficients (P), by using the hyperprior distribution of the variance-covariance matrix S. |
| ***Likelihood:***  For(i in 1:Number of Observations) 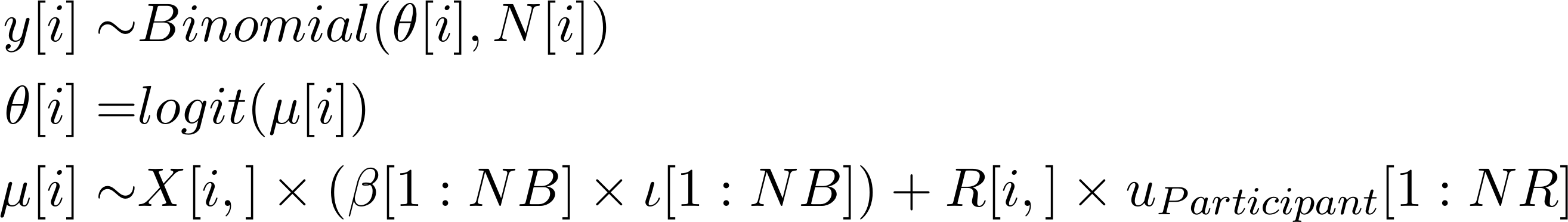 | | *y[i]* is the dependent variable, the MVP accuracy. This is distributed according to a binomial distribution, with probability of being correct θ*[i]* and the total number of observations *N[i]*. The θ*[i]* parameter is given by the application of the logit function to the *µ[i]* parameter, that is given by the linear combination of the fixed and random coefficients from the model.  The fixed coefficients of the model are multiplied by the ι parameter, which can force to zero the coefficients of a factor, which will not be used to take into account for the data. |

### Table SM4. Binomial models with PSM to test random answers.

In this simple model we only wanted to test if the performances in the Movement Verbal Feedback (MVP) were correct (probability to give a correct answer of 0.85), or there was no difference with random responses (probability of 0.50 to give a correct answer).

Description as in SM3.

| ***Priors:*** 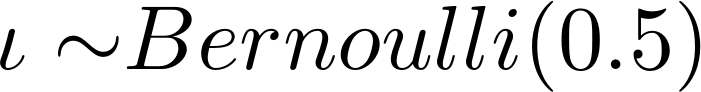 | The prior distribution for θ*[i]* is a non-informative Bernoulli distribution which can take the value of 0 or 1 with the same probability. |
| --- | --- |
| ***Likelihood:***  For(i in 1:Number of Observations) 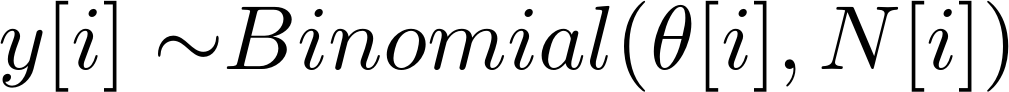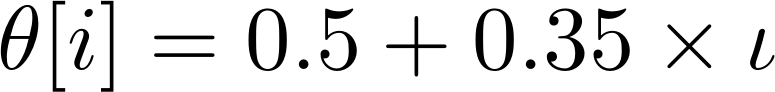 | *y[i]* is the dependent variable, the MVP accuracy. This is binomially distributed with probability of giving a correct answer θ*[i]* = 0.5 with ι =0 (0.5+0.35*(ι=0) = 0.5) or θ*[i]* = 0.85 with ι =0 (0.5+0.35*(ι=1) = 0.85). |

### Table SM5. Linear models with PSM.

Bayesian linear models for physiological and VAS analyses. The data distribution is gaussian; for this reason we used Bayesian multilevel models for gaussian data (Gelman and Hill, 2006; Kruschke, 2014).

The PSM is given by the estimation of ι index (that can take 0 or 1 values) for each factor. This gives the probability of that factor to be used to explain the data variability.

“NR” is the number of the random coefficients, “NB” is the number of the fixed effects, “b” is the index for parameters that are connected with fixed effects, “r” is the index for parameter that are connected with random effects, “f” is the index for the factors and their interactions, “i” is the index for the dependent variable. “X” represents the matrix for fixed effects, “R” the matrix for random effects, “P” the matrix for the correlations among random coefficients, “S” the variance-covariance matrix among random coefficients. The “β” are the coefficients for the fixed effects, with parameters “µ” (mean) and “σ” (standard deviation). The “u” are the coefficients for the random effects, with parameters “ϖ” (mean) and “ς” (standard deviation). The “ϑ” coefficient is the variance for the likelihood. The “shape” and “rate” parameters for the gamma distributions, used as hyperpriors for the “σ” and “ϑ” coefficients, and they are equal to shape = SD(y)/2 and rate = 2SD(y) (Kruschke, 2014 p.561).

| ***Hyperpriors:***  For(b in 1:Number of Fixed Coefficients) 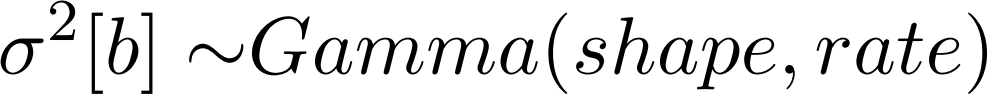 For(r in 1:Number of Random Coefficients) 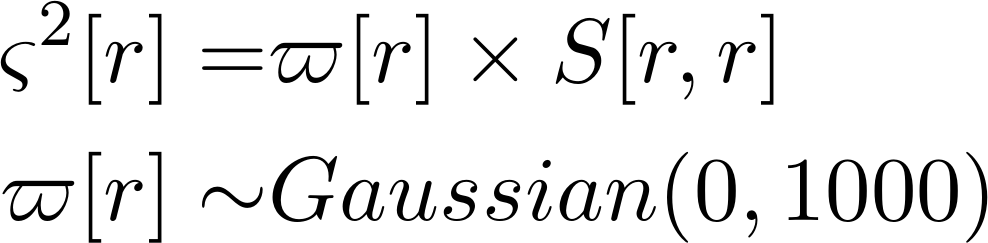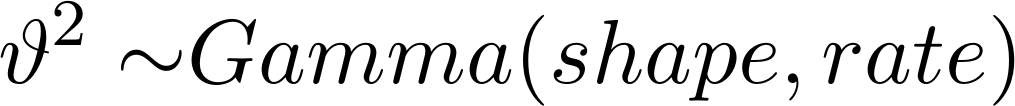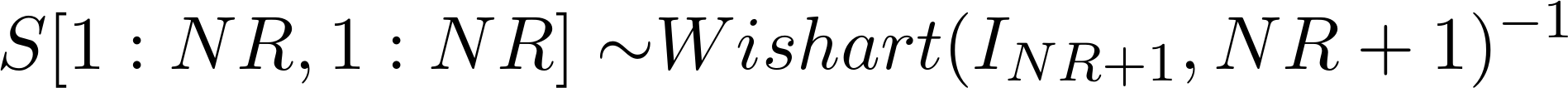 | The hyperprior distributions are non-informative (therefore no prior assumptions are taken on them). We determine the variance for the priors for the fixed coefficients (σ^2^), the mean and variance parameters for the random coefficients ($\varpi$and $\varsigma^{2}$), the common variance of the model (ϑ^2^) and the variance-covariance matrix for the random coefficients (S).  The fixed coefficients are the coefficients used to take into account the fixed factors, namely the experimentally manipulated factors. The random coefficients are the coefficients used to take into account the random variation that is not experimentally manipulated, such as the individual ability, learning effects or fatigue effects. |
| --- | --- |
| ***Priors:***  For(b in 1:Number of Fixed Coefficients) 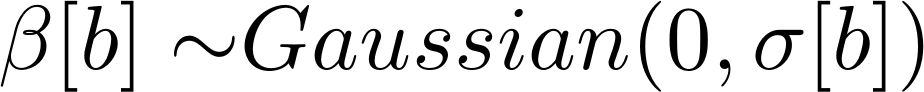 For(f in 1: Number of Factors) 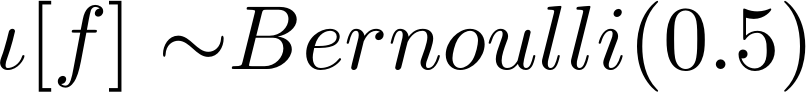 For(r in 1:Number of Random Coefficients) 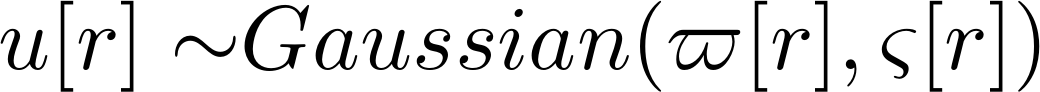 For(r1 in 1:Number of Random Coefficients)  For(r2 in 1:Number of Random Coefficients) 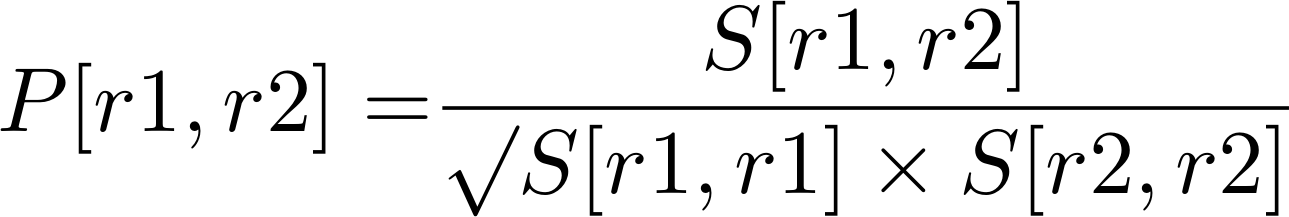 | The prior distributions for the fixed, random and ι coefficients of the model.  The fixed effects coefficients (β) are determined by non-informative distributions.  The ι index may take values within the [0÷1] range for all the factors from a non-informative distribution. This index is then adapted to take into account all the coefficients for each factor. When ι=1 in a single iteration, the factor is used for accounting for the data in that iteration.  Also, the random coefficients (*u*) are determined via non-informative distributions, whose parameters are the hyperprior distributions of *ϖ* and *ς*.  Here we also determine the correlation matrix among random coefficients (P), by using the hyperprior distribution of the variance-covariance matrix S. |
| ***Likelihood:***  For(i in 1:Number of Observations) 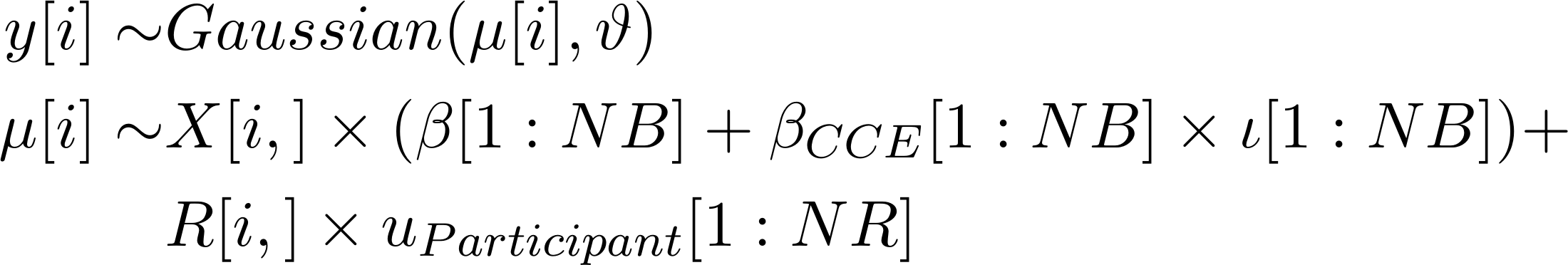 | Likelihood distribution for the dependent, Gaussianally distributed, variable *y*.  The ι distribution may force to zero the β coefficients of a single factor, taking the value of zero. |

## SM2. Bayesian analyses for SCL and RSA data:

In analysis of SCL the fixed factors were Group (C, CP, IP) and Condition (No Stimulation, Vision: Mobilization – Motion: Mobilization, Vision: Mobilization – Motion: No Mobilization, Vision: No Mobilization – Motion: Mobilization, Vision: No Mobilization – Motion: No Mobilization). The random effects were the within-subjects factor Condition and the numerical order of the blocks converted in z-scores.

Results showed inconclusive results for the main effects of Group (BF_10_ =0.37) and Condition (BF_10_ =0.92), and a null effect for the interaction (BF_10_ =0.10).

The analyses of RSA used the same fixed and random effects. Results showed inconclusive results for the main effect of Group (BF_10_ =2.45) and Condition (BF_10_ =0.90), and a null effect for their interaction (BF_10_ =0.005).

## References

Brooks SP, Gelman A (1998) General Methods for Monitoring Convergence of Iterative Simulations General Methods for Monitoring Convergence of Iterative Simulations. J Comput Graph Stat 7:434–455.

Carlin BP, Chib S (1995) Bayesian model choice via Markov Chain Monte Carlo method. J R Soc B 57:473–484.

Gelman A, Hill J (2006) Data Analysis Using Regression and Multilevel/Hierarchical Models. Available at: http://books.google.it/books/about/Data_Analysis_Using_Regression_and_Multi.html?id=c9xLKzZWoZ4C&pgis=1 [Accessed January 12, 2015].

Gelman A, Rubin DB (1992) Inference from Iterative Simulation Using Multiple Sequences. Stat Sci 7:457–472.

Geman S, Geman D (1984) Stochastic relaxation, Gibbs distributions, and the Bayesian restoration of images. Pattern Anal Mach Intell IEEE Trans:721–741.

Kellner K (2017) jagsUI: A Wrapper Around “rjags” to Streamline “JAGS” Analyses. Available at: https://cran.r-project.org/package=jagsUI.

Kruschke JK (2014) Doing Bayesian data analysis: A tutorial with R, JAGS, and Stan, second edition, 2nd ed. Elsevier Inc. Available at: http://dx.doi.org/10.1016/B978-0-12-405888-0.09999-2.

Lewis SM, Raftery AE (1997) Estimating Bayes Factors via Posterior Simulation with the Laplace—Metropolis Estimator. J Am Stat Assoc 92:648–655 Available at: http://www.tandfonline.com/doi/abs/10.1080/01621459.1997.10474016.

Lodewyckx T, Kim W, Lee MD, Tuerlinckx F, Kuppens P, Wagenmakers E-J (2011) A tutorial on Bayes factor estimation with the product space method. J Math Psychol 55:331–347 Available at: http://linkinghub.elsevier.com/retrieve/pii/S0022249611000423 [Accessed August 26, 2015].

Maravita A, Spence C, Kennett S, Driver J (2002) Tool-use changes multimodal spatial interactions between vision and touch in normal humans. Cognition 83:B25-34 Available at: http://www.ncbi.nlm.nih.gov/pubmed/11869727.

Pavani F, Spence C, Driver J (2000) Visual capture of touch: Out-of-the-body experiences with rubber gloves. Psychol Sci 11:353–359 Available at: http://www.ncbi.nlm.nih.gov/pubmed/11228904 [Accessed October 7, 2013].

Pinheiro JC, Bates DM (2000) Mixed-Effects Models in S and S-Plus. Springer. Available at: http://books.google.co.uk/books/about/Mixed_Effects_Models_in_S_and_S_PLUS.html?id=N3WeyHFbHLQC&pgis=1 [Accessed January 12, 2015].

Plummer M (2015) JAGS: A program for analysis of Bayesian graphical models using Gibbs sampling, ver 3.3.3. Available at: http://mcmc-jags.sourceforge.net/.

R Core Team (2017) R: A Language and Environment for Statistical Computing. Available at: http://www.r-project.org.
